# Sex-Based Differences in Mitral Annular Disjunction Severity and Arrhythmic Risk in Mitral Valve Prolapse

**DOI:** 10.1016/j.echo.2025.08.026

**Published:** 2025-09-15

**Authors:** Luca Cristin, Lionel Tastet, Rohit Jhawar, Amy B. Rich, Janet J. Tang, Dwight Bibby, Qizhi Fang, Farzin Arya, Francesca N. Delling

**Affiliations:** Department of Medicine (Cardiovascular Division), University of California, San Francisco, California.

**Keywords:** Arrhythmia, Mitral annular disjunction, Mitral valve prolapse, Sex-based differences

## Abstract

**Background:**

Arrhythmic mitral valve prolapse (AMVP), a condition with known female predominance, has been linked to mitral annular disjunction (MAD), yet the relationship between MAD, sex, body size, and arrhythmic events remains unclear.

**Objectives:**

To determine whether MAD exhibits sex-based differential effects on arrhythmic risk and explore the implications of indexing MAD measurements.

**Methods:**

We examined 682 consecutive MVPs at the University of California San Francisco (2013–23) with detailed clinical, rhythmic, and echocardiographic data. Mitral annular disjunction was indexed to body surface area, resulting in the identification of 3 distinct groups: no MAD and indexed MAD (iMAD) below and iMAD above the median. Arrhythmic mitral valve prolapse was defined as MVP with frequent and/or complex ventricular ectopy, including severe arrhythmic events such as sustained ventricular tachycardia, ventricular fibrillation/sudden cardiac arrest, or death.

**Results:**

Among 682 MVP patients (48% women, mean age 58 ± 17 years), 35% had AMVP and 41% had MAD, with a median iMAD of 4 mm. Compared to women, men had greater absolute MAD length (9.0 ± 3.2 mm vs 7.3 ± 2.6, *P* < .001). Sex-based differences in MAD length were no longer appreciated after indexing for body surface area (*P* = .33). In multivariable analyses, iMAD >4 mm was significantly associated with arrhythmic events in men but not in women (*P* = .02 vs *P* = .63). Bileaflet MVP had a direct effect on severe arrhythmic events in women (*P* = .04 for MVP, *P* = .39 in mediation analysis), but in men this association was mediated by iMAD length (*P* = .03).

**Conclusion:**

In a large MVP cohort, men exhibit greater MAD length and a stronger association between iMAD length and arrhythmic events. In contrast, iMAD length in women may be less important for arrhythmic risk stratification compared to other imaging parameters such as bileaflet involvement. Further studies are needed to confirm alternative mechanisms beyond localized MAD-related myocardial traction in women with MVP.

## Central Illustration

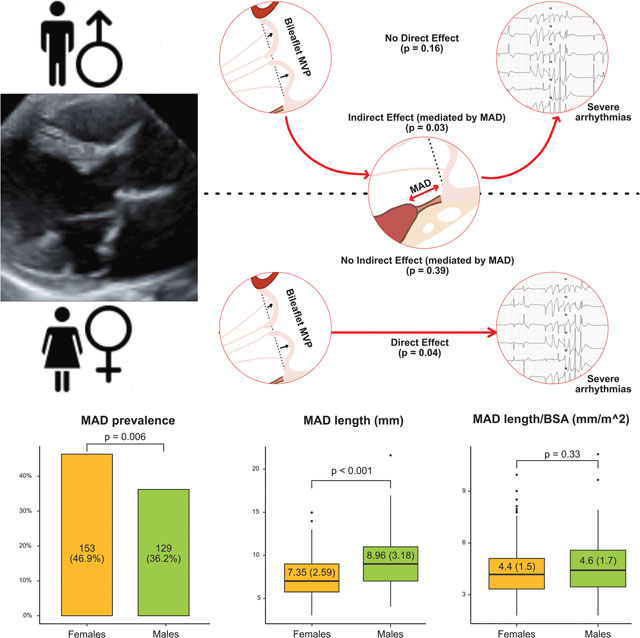

Mediation analyses for the development of severe arrhythmia in MVP patients according to sex. Sex-based differences in the pathways through which bileaflet MVP affects severe arrhythmic outcomes, with MAD as a mediator. In men (*upper section*) bileaflet MVP without MAD leads to no observable severe arrhythmic events (no direct effect), whereas the presence of MAD results in an indirect effect on arrhythmia. Conversely, the *lower section*, representing women, shows that bileaflet MVP directly impacts severe arrhythmic outcomes without mediation by MAD (direct effect), and there is no indirect effect mediated by MAD. The panel below shows a comparison of MAD prevalence between women and men, with box plots illustrating the differences between sexes in absolute MAD and iMAD length.

## INTRODUCTION

Mitral valve (MV) prolapse (MVP) is a common valvulopathy affecting 2% to 3% of the general population.^1–3^ While most cases of MVP are relatively benign, a small yet nontrivial proportion of individuals can develop malignant ventricular arrhythmias and sudden cardiac death (SCD) independently of the severity of mitral regurgitation (MR).^4–8^ Arrhythmic MV prolapse (AMVP) has been associated with a bileaflet phenotype, focal fibrosis in the papillary muscles or basal inferolateral left ventricular (LV) myocardium, and mitral annular disjunction (MAD).^9^

Mitral annular disjunction is defined as a separation between the left atrial (LA) wall at the level of the MV junction and the basal LV myocardium.^10^ Mitral annular disjunction is postulated to exacerbate MVP-related abnormal valvular-myocardial mechanics by causing hypermobility of the MV apparatus and increasing myocardial traction by the prolapsing leaflets, which in turn can lead to ventricular arrhythmias.^11,12^ Although AMVP is a condition with known female predominance,^6,13^ sex-based differences in MAD prevalence and length have not been investigated. Moreover, the impact of MAD length when considering body size is unclear. Notably, the length of MAD, like all cardiac dimensions, may vary according to heart size and sex-specific anatomical differences. As for other echocardiographic parameters, indexing to body surface area (BSA) allows standardization and improves comparability across individuals of varying body sizes and different sex. Some studies suggest an association between MAD length and arrhythmic risk. However, specific MAD length cutoffs for increased risk have been inconsistent.^12,14–17^ We hypothesize that such conflicting results may arise from an underappreciation of sex-based differences in the impact of MAD on arrhythmic events. Finally, previous studies focused on the association of MAD with ventricular arrhythmias have not controlled for bileaflet MVP, another common risk factor for AMVP. Not all AMVP patients with MAD have a bileaflet phenotype,^12,18^ nor do all bileaflet AMVPs have MAD,^18^ suggesting that MAD and bileaflet MVP can act independently of each other as determinants of arrhythmic risk.

Our study aims to (1) investigate sex-based differences in MAD length and the implications of indexing MAD length to body size in individuals with MVP; (2) determine whether MAD length exhibits a sex-based differential effect on arrhythmic risk; and (3) understand whether bileaflet MVP and MAD, which are often (but not consistently) found in association particularly in women, can act independently of each other on the risk of arrhythmic events.

## METHODS

### Study Population

We identified 682 patients with MVP who underwent comprehensive Doppler echocardiography as part of clinical care at the University of California San Francisco (UCSF) from 2013 to 2023. Eligibility for inclusion was determined based on the availability of transthoracic echocardiographic images for each patient, which allowed for a comprehensive evaluation of LV morphology and function, MV characteristics, including the assessment of MAD presence and length, and speckle-tracking echocardiography parameters. Patients were excluded if they had a previous MV intervention (i.e., repair or replacement), other valvulopathies greater than mild, or—pertaining to the arrhythmic end point analysis—a history of coronary artery disease. The latter was defined as a prior myocardial infarction or percutaneous/surgical coronary intervention. In addition, patients with concomitant arrhythmogenic conditions such as hypertrophic cardiomyopathy, sarcoidosis, arrhythmogenic right ventricular cardiomyopathy, and idiopathic dilated cardiomyopathy were excluded. Clinical, rhythmic, and echocardiographic data were retrieved from medical records and organized in an electronic database as part of a UCSF MVP registry. Clinical and arrhythmic events were continuously assessed over time following the baseline echocardiogram. The UCSF Institutional Review Board approved the study and waived informed consent.

### Clinical and Rhythmic Data

Clinical data included age, sex, race, BSA, heart rate, and blood pressure. Clinical comorbidities were also recorded.

Arrhythmic mitral valve prolapse was defined based on a consensus statement by the European Heart Rhythm Association^9^ as MVP with (1) history of sudden cardiac arrest (SCA)/SCD, (2) complex ventricular ectopy, including sustained or nonsustained ventricular tachycardia and ventricular fibrillation, or (3) frequent (≥5%) premature ventricular contractions in the absence of other well-defined arrhythmic substrates. A severe arrhythmic event was defined as the occurrence of ventricular fibrillation, SCA, SCD, and/or sustained ventricular tachycardia (VT). Rhythmic data were obtained through comprehensive chart review, ambulatory electrocardiogram (ECG) monitoring (Holter or event monitor), inpatient telemetry, Emergency Medical Services records, or the National Death Index for SCD cases. The median follow-up duration was 5 years (interquartile range, 3–7) from the date of the baseline echocardiogram.

### Echocardiographic Evaluation

Echocardiographic measurements, including MAD length, LV volumes, LV mass, and strain parameters, were retrospectively performed from stored images by an experienced research sonographer (F.A.) and reviewed by a board-certified physician with expertise in MVP imaging (F.N.D.). Two-dimensional transthoracic echocardiograms were performed using commercially available ultrasound systems. Mitral valve prolapse was defined as superior displacement of 1 or both mitral leaflets ≥2 mm beyond the mitral annulus in a parasternal or apical 3-chamber long-axis echocardiographic view.^19^ The MVP phenotype was further subdivided in flail, bileaflet, or single-leaflet prolapse (anterior vs posterior). The severity of MR was determined using a multiparametric approach, as recommended by guidelines, and classified as none/trace or mild, moderate, or severe.^20^ Mitral annular disjunction was defined as a separation between the LA/MV junction and the inferolateral basal myocardium and was measured in the parasternal long-axis view.^7,21^ Cases in which a portion of the posterior leaflet abutted the LA wall in systole without a true separation from the myocardium were excluded by carefully assessing leaflet insertion frame-by-frame.^22^ Mitral annular disjunction was then indexed to BSA (iMAD). Patients were further categorized into 3 groups: no MAD and iMAD below and above the median of the whole cohort.

According to the recommendations of the American Society of Echocardiography and the European Association of Cardiovascular Imaging, the LV end-diastolic and end-systolic volumes, LV mass, and LV ejection fraction (LVEF) were indexed to BSA.^23^ Right ventricular systolic function was semiquantitatively assessed by visual examination and classified in normal, mildly reduced, and severely reduced.

Speckle-tracking echocardiography analysis was performed offline using a commercially available software (TOMTEC ARENA) to measure LV global longitudinal strain (GLS), a myocardial parameter known to be affected by severe MR.^24^ Left ventricular GLS was defined as the percentage of longitudinal shortening, calculated as the change in length relative to the baseline length.^23^ This value was obtained by averaging segmental measurements from 3 standard apical views. We also measured mechanical dispersion (MD), defined as the SD of the time to peak longitudinal strain across the 16 standard myocardial segments.

To assess inter- and intraobserver reproducibility for MAD length, a subgroup of 20 patients was independently reevaluated by a second observer (L.C.), blinded to MAD measurements and clinical outcomes. Agreement was quantified using the intraclass correlation coefficient. For the interobserver variability, the intraclass correlation coefficient was 0.42 (95% CI, 0.013–0.712; *P* = .02), indicating moderate but statistically significant consistency. For the intraobserver variability, the intraclass correlation coefficient was 0.93 (*P* < .001), indicating excellent consistency.

### Study End Points

The primary end points of the study were (1) presence of MAD (absolute and indexed to BSA; (2) AMVP (i.e., the composite of frequent/complex ventricular ectopy and SCA/SCD) and within the AMVP group, and (3) occurrence of the most severe arrhythmic presentations (sustained VT and SCA/SCD).

### Statistical Analyses

Continuous variables with normal distribution are presented as means ± SD and were compared using the Student’s *t*-test. Nonnormally distributed continuous variables are presented as medians with interquartile ranges and were compared using the Kruskal-Wallis test. Categorical variables are presented as frequencies and percentages and were compared with the*χ*^2^ test or Fisher’s exact test as appropriate.

Univariable and multivariable logistic and linear regression analyses were performed to identify the predictors of MAD presence and length. The multivariable model included all clinically relevant variables and those significantly associated (i.e., *P* < .05) in univariable analysis. Results are presented as odds ratios (ORs) and 95% CIs or standardized beta coefficient (*ß*) with standard error as appropriate. In the univariable and multivariable logistic regression analyses for arrhythmia predictors, iMAD was analyzed as both a continuous and a categorical variable. For the continuous analysis, iMAD values were imputed as 0 for patients without MAD. A mediation analysis was used to test the potential role of MAD as a mediator in the relationship between bileaflet MVP and severe arrhythmic events.^25,26^ The CIs for the average causal mediation effect, average direct effect, and the proportion mediated were computed using a quasi-Bayesian approach with 1,000 draws.^25^ To account for potential confounding in sex-based comparisons, 1:1 propensity score matching was performed using nearest-neighbor matching with a caliper of 0.05. Matching variables included hypertension, atrial fibrillation, indexed LV end-diastolic volume, end-systolic volume, and LV mass index. Covariate balance was assessed using standardized mean differences and eCDF metrics.

A 2-tailed *P* < .05 was considered statistically significant. Statistical analyses were performed using RStudio (RStudio Team [2020], ver. 2023.12.1 + 402).

## RESULTS

### Study Population

There were 682 patients with a diagnosis of MVP and MAD evaluation (326 women; mean age, 58 ± 17 years). Clinical and echocardiographic characteristics of the study population are presented in Table 1. Bileaflet MVP was present in 304 (45%), and flail leaflet in 43 (6%). Mitral regurgitation was mild or less in 66% of cases, moderate in 25%, and severe in 9%. Mitral annular disjunction was diagnosed in 282 patients (41%), with a mean absolute length of 8.1 ± 3.0 mm. The median of iMAD in the overall population was 4.3 mm (rounded to 4 mm). Based on iMAD length, patients were categorized into 3 groups: no MAD, iMAD below the median, and iMAD above the median. In total, 19% of MVPs had an iMAD greater than 4 mm. In the entire cohort, 34% of patients met the criteria for arrhythmic MVP. Specifically, 29% had nonsustained ventricular tachycardia documented on ambulatory ECG monitoring or electrophysiology clinic notes, 5% had frequent PVCs, and 4% experienced a severe arrhythmic event. Among the 27 with severe arrhythmic events, there were 16 cases of SCA with subsequent implantable cardioverter-defibrillator placement for secondary prevention, 4 SCD cases, and 7 cases of sustained VT.

Table 1 shows the study characteristics stratified by sex. Female patients had significantly lower BSA and lower diastolic blood pressure and were less likely to have hypertension and atrial fibrillation (all *P* < .001). When considering echocardiographic characteristics, women had lower indexed LV end-systolic and end-diastolic volumes compared to men, with similar LVEF, MR severity, and LV GLS. Moreover, female patients had a higher prevalence of MAD (47% vs 36%, *P* = .006), but mean MAD length was significantly lower (7.4 mm vs 9.0 mm, *P* < .001). Sex-based differences in MAD length were no longer appreciated after indexing for BSA (iMAD: 4.4 ± 1.5 mm in women vs 4.6 ± 1.7 mm in men; *P* = .33; Table 1). Compared to non-AMVPs, AMVPs were commonly White and had a higher prevalence of MAD and atrial fibrillation but were less hypertensive and had slightly higher indexed LV end-diastolic volume (all *P* < .01; Supplemental Table 1). In the overall population, MAD was significantly associated with AMVP both when analyzed as a binary variable (*P* = .01) and as a continuous indexed measurement (*P* = .04), even after adjusting for covariates that were significant in univariable analyses (Supplemental Table 2). However, when iMAD was categorized based on its median value, the association was no longer statistically significant (Supplemental Table 2).

### Factors Associated with MAD Presence and Length

Multivariable analyses demonstrated that female sex was an independent predictor of the presence of MAD, irrespective of age, race, or bileaflet involvement (*P* = .01; Table 2). Conversely, male sex was independently associated with greater absolute MAD length (*ß* = 0.82 × 0.39, *P* = .04), after adjusting for age, bileaflet MVP, and indexed LV volumes and mass (Table 2). To further account for potential confounding, we performed 1:1 propensity score matching between men and women based on hypertension, atrial fibrillation, indexed LV end-diastolic and end-systolic volumes, and LV mass index. Even after matching, male patients continued to exhibit significantly longer absolute MAD length (mean difference, 1.31 ± 0.65 mm, *P* = .0465). However, when MAD length was indexed to BSA (iMAD), the difference was no longer significant (*P* = .963; Supplemental Table 3).

### Sex-Specific Predictive Value of MAD Length in Arrhythmic MVP

The presence of MAD was significantly associated with AMVP in men after adjusting for age, MR severity, and bileaflet involvement (OR = 2.04; 95% CI, 1.12–3.46; *P* = .01). Conversely, in women, the association between MAD presence and AMVP was significant only in univariable analysis and did not persist after multivariable adjustments for the same covariates (*P* = .27; Table 3). In contrast, bileaflet MVP showed a significant association with AMVP (*P* = .04; Table 3).

Similar sex-specific differences were observed regarding MAD length. In men, greater iMAD length (i.e., median iMAD >4 mm) was associated with an increased risk of AMVP, independently of MR and bileaflet MVP (OR = 2.2; 95% CI, 1.1–4.2; *P* = .02; Table 3). In contrast, in women, greater iMAD was not a significant predictor of AMVP, whereas bileaflet involvement was associated with AMVP risk (*P* = .04; Table 3). After adjusting for age, bileaflet MVP, and MR severity, iMAD as a continuous variable was associated with AMVP in men (OR = 1.15; 95% CI, 1.04–1.28; *P* = .01), but not in women (OR = 1.06; 95% CI, 0.96–1.17; *P* = .27), as shown in Table 3. Within the AMVP group, iMAD as a continuous variable predicted severe arrhythmic events (including sustained VT, ventricular fibrillation/SCA, and SCD) only in men (OR = 1.24 [1.02–1.51], *P* = .03) and not in women (*P* = .50). In contrast, MD was significantly associated with AMVP only in women (OR = 1.02; 95% CI, 1.00–1.03; *P* = .02), while no association was observed in men (*P* = .76; Supplemental Table 4).

### Sex-Specific Role of MAD Length in Bileaflet MVP-Induced Severe Arrhythmic Events

Table 4 shows the results of a mediation analysis aimed at understanding how the relationship between bileaflet MVP and severe arrhythmic events differs by sex and is mediated by iMAD length. In men, bileaflet MVP had a significant indirect effect on severe arrhythmic risk through iMAD (Table 4, *P* = .002), with 41% of the total effect mediated (proportion mediated = 0.41; 95% CI, 0.11–1.51; *P* = .03) by iMAD. There was no significant direct effect of bileaflet MVP on severe arrhythmic events in men (*P* = .16). In contrast, in women, there was a direct effect of bileaflet MVP on severe arrhythmic events (Table 4, *P* = .04), without significant mediation by iMAD length (*P* = .39; Central Illustration).

## DISCUSSION

In a large, unselected MVP cohort we demonstrate that (1) MAD presence is associated with female sex independently of bileaflet MVP and MR severity; (2) men have greater absolute MAD length; however, after indexation, sex-based differences in MAD length are no longer observed; (3) iMAD length is associated with severe arrhythmic events only in men; (4) in men, the effect of bileaflet MVP on arrhythmogenesis is only indirect (mediated by MAD), while in women, the effect is direct and not mediated by MAD.

Patients with MVP without severe MR or LV dysfunction have an overall survival comparable to that of the general population.^27^ However, certain factors—such as severe myxomatous disease in bileaflet MVP, excessive leaflet length and thickness, and MAD, irrespective of MR severity—have been associated with a higher risk of arrhythmic events.^28^ In our study, we report for the first time sex-based differences in the contribution of mitral morphofunctional characteristics to arrhythmic risk, emphasizing the significance of indexing to body size to enhance understanding of the underlying mechanisms for AMVP.

### MAD and Arrhythmic MVP

The association between MAD and AMVP has been documented in various studies, although discrepancies exist.^15,16,18,29^ In a large cohort of MVP patients, Essayagh *et al.*^18^ demonstrated that MAD presence is independently associated with ventricular arrhythmias, albeit not with higher mortality. Mitral annular disjunction contributes to arrhythmogenesis by leading to a partial loss of mechanical annular function, thus augmenting the traction exerted by the prolapsing leaflets on the myocardium.^12,15,18^ However, not all patients with MAD develop ventricular arrhythmias, and our recent postmortem study indicates that SCD in MVP can occur without MAD.^8^ Additional factors must be considered to fully understand the relationship between MAD and arrhythmic risk in MVP.

In our cohort, MAD was independently associated with AMVP in the overall population when analyzed as a binary or continuous indexed variable (iMAD; Supplemental Table 2). However, this association was attenuated when iMAD was categorized by its median value, likely reflecting the sex-specific differences observed in our stratified analyses.

### Sex-Based Differences in MAD Presence and Length

In our large cohort of unselected MVP patients, female sex was associated with the presence of MAD independently of bileaflet phenotype, age, and race (OR = 1.57; CI, 1.13–2.19; *P* = .01; Table 2). These findings are consistent with the results of a previous study on 90 MVP patients.^30^ However, in a larger cohort of patients, Essayagh *et al.*^18^ found that female sex was not associated with MAD. This discrepancy may be due to the complex interrelationship between bileaflet MVP, female sex, and MAD, which was not addressed in prior investigations.

In our study, absolute MAD length was associated with male sex both at the univariable level and after adjustment for age, MR severity, bileaflet phenotype, and indexed LV end-diastolic volume and mass (*ß* = 0.82 ± 0.39; *P* = .04). This association may be attributed to the difference in heart size between men and women or other sex-related intrinsic characteristics.^31^ Men generally have larger hearts than women, making it crucial to index echocardiographic measurements to body size to ensure accurate comparisons.^31,32^ Indexing measurements to body size is essential to establish limits of normality, correlate abnormal values to outcomes, and select the most appropriate timing for surgical or percutaneous intervention. According to American Society of Echocardiography/European Association of Cardiovascular Imaging guidelines, echocardiographic measurements should be indexed to body size, typically represented by BSA.^33^ After indexation, the sex-based difference in MAD length was no longer significant (*P* = .27), suggesting that the initially observed difference was primarily due to heart size rather than sex.

### Indexed MAD Length and Its Impact on Arrhythmic Risk in MVP

Recent studies have highlighted the abnormal valvular-ventricular interactions capable of altering ventricular biology and rhythm, particularly in bileaflet MVP. These include papillary muscle traction by the prolapsing leaflets,^34^ associated curling motion of the basal inferolateral LV, and systolic annular expansion, which can increase the force exerted on the LV.^35–38^ Such valvular-ventricular interactions are thought to be augmented by MAD; however, the predominant role of MAD in bileaflet MVP remains unclear. Moreover, differences in sex and the interplay between bileaflet MVP and MAD, which often coexist, have not been thoroughly investigated.

In our large cohort of MVP patients, we analyzed MAD presence and length independently of bileaflet phenotype and according to sex. Our results showed that in men, MAD length is independently associated with AMVP, while in women, this association is not as strong. Moreover, a subanalysis aimed at understanding, among those with ventricular arrhythmias, the risk of developing the most severe arrhythmic events, revealed that after adjusting for bileaflet MVP, MR severity, and age, MAD length as a continuous variable was associated with severe arrhythmic events only in men (OR = 1.24 [1.02–1.51], *P* = .03), but not in women (*P* = .5). Left ventricular ejection fraction and LV GLS, parameters affected by significant MR, were not significantly different between the AMVP and non-AMVP groups,^39,40^ suggesting that MR and MR-related myopathy were not the primary drivers of arrhythmic events. It is important to note that an iMAD ≥4 mm, which we used as a threshold in our risk analyses, likely reflects a pathologic degree of disjunction that is uncommon in healthy individuals. Previous studies using more sensitive imaging modalities have reported normal absolute MAD values around 2 to 3 mm in control populations.^41^ Sex-based differences in the association between MAD and ventricular arrhythmias may explain the lack of consensus regarding the role of MAD length in arrhythmic MVP.^12,14–17^

### MAD versus Bileaflet MVP: Sex-Based Differences in Arrhythmic Risk

Mitral annular disjunction and bileaflet MVP often coexist, further complicating our understanding of valvular-myocardial mechanics and arrhythmogenesis. A recent study by Perazzolo Marra *et al.*^29^ identified curling motion as a key arrhythmogenic marker in bileaflet Barlow disease, exerting both a direct mechanical effect and an indirect effect mediated by myocardial fibrosis. However, their analysis did not stratify by sex or isolate the respective roles of MAD and bileaflet MVP. For the first time, we demonstrate that MAD and bileaflet MVP may act differently on arrhythmic risk depending on sex: in men, the indexed length of MAD is associated with AMVP, independently of the presence of bileaflet MVP. However, this is not observed in women. Moreover, MD, a marker of heterogeneous contraction and electrical dispersion, was significantly associated with AMVP only in women.^42^ This finding further supports a sex-specific substrate where a diffuse, primary myopathy may have a greater role in women compared to men. Through an exploratory mediation analysis, we demonstrate that bileaflet MVP does not directly cause severe arrhythmic events in men; instead, it influences arrhythmogenesis indirectly through MAD. In contrast, in women, there is a significant direct relationship between bileaflet MVP and severe arrhythmic events, without any mediation by MAD (Central Illustration). These findings offer preliminary insights into potential sex-specific mechanistic pathways that warrant further longitudinal investigation. These results suggest that the pathophysiology of arrhythmic complications in women with MVP is more complex and is not solely dependent on MAD-related abnormal myocardial traction. A primary (i.e., genetic) myopathy has been proposed by our group and others as a potential “substrate” for arrhythmogenesis.^43–45^ Histopathological differences, including variations in myocardial fibrosis patterns, fibrous disjunction, or differences in extracellular matrix composition, could influence how men and women respond to valvular mechanical stress. Indeed, our group recently demonstrated a more diffuse pattern of fibrosis in women with AMVP using cardiac magnetic resonance (CMR)/T1 mapping.^46^ In this context, even lesser degrees of myocardial traction (bileaflet MVP without MAD), or no traction (minimal MVP), could translate into arrhythmic events in genetically predisposed individuals.^8^ Further studies are required to elucidate alternative arrhythmic mechanisms beyond pure abnormal myocardial traction in both sexes, and particularly in women with bileaflet MVP.

## LIMITATIONS

The present study is a retrospective single-center analysis and is subject to inherent limitations related to its cross-sectional design. While arrhythmic data were evaluated over time from the date of the baseline echocardiogram, there was no standardized longitudinal follow-up with scheduled ambulatory ECG monitoring. Therefore, we opted not to pursue time-to-event analyses. Nevertheless, clinical and arrhythmic events were captured through comprehensive chart review over a median observational period of 5 years. This extended temporal gap between the baseline echocardiogram and subsequent event ascertainment may weaken the direct linkage between static imaging findings and evolving arrhythmic risk; thus, our statistical inferences relating echocardiographic measures to outcomes should be interpreted with caution. However, lack of standardized arrhythmic surveillance reflects “real world” clinical practice, where guidelines regarding exact timing of repeat ambulatory ECG monitoring are unavailable.

Although CMR is considered the gold standard for structural characterization and detection of myocardial fibrosis, CMR data were available only for a limited number of patients and were therefore not reported. As a result, we focused exclusively on echocardiographic parameters.

The measurement of MAD was not performed in the context of a Corelab; however, the review and the analyses of echocardiographic images were performed by experienced readers blinded to outcome data. The measurements of MAD and other echocardiographic parameters were made in accordance with previous studies. We note, however, that annular dimensions such as diameter and area were not captured, limiting our ability to assess the full spectrum of mitral apparatus geometry. In addition, MAD demonstrated only moderate interobserver reproducibility. However, intraobserver agreement was excellent. Mitral annular disjunction was relatively common in our study, although its prevalence was similar to that reported in other studies.^28^ Importantly, this is the first study to index MAD measurements to body size and correlate them with arrhythmic risk. Furthermore, our findings support a novel hypothesis about different arrhythmogenic pathways in men and women with MVP. These results should be considered hypothesis-generating and warrant validation in future prospective, multicenter studies with standardized imaging protocols, addition of CMR data, and systematic rhythmic follow-up.

## CONCLUSION

In a large, unselected MVP cohort, men exhibit greater MAD length and a stronger association between iMAD and arrhythmic events. In contrast, iMAD length in women may be less important for arrhythmic risk stratification compared to other imaging parameters such as bileaflet involvement or MD. Our findings highlight the importance of considering sex-specific differences in the assessment and management of arrhythmic risk in patients with MVP and MAD. Further studies are required to elucidate alternative arrhythmic mechanisms beyond pure MAD-related abnormal myocardial traction in women with bileaflet MVP.

## Supplementary Material

1

SUPPLEMENTARY DATA

Supplementary data related to this article can be found at https://doi.org/10.1016/j.echo.2025.08.026.

## Figures and Tables

**Table 1:** Characteristics of the study population stratified by sex

	All patients (*N* = 682)	Female patients (*N* = 326; %)	Male patients (*N* = 356, %)	*P* value

Clinical characteristics				
Age, years	58 (17)	58 (17.)	59 (16)	.447
Non-White race, *n* (%)	183 (27)	80 (25)	103 (29)	.228
BSA, m^2^	1.8 (0.3)	1.7 (0.26)	2.0 (0.2)	**<.001**
Systolic blood pressure, mm Hg	126 (50)	126 (70)	127 (17)	.722
Diastolic blood pressure, mm Hg	69 (10)	67 (10)	71 (10)	**<.001**
Heart rate, bpm	71 (22)	72 (26)	70 (16)	.263
Hypertension, *n* (%)	256 (38)	93 (29)	163 (46)	**<.001**
Hyperlipidemia, *n* (%)	170 (25)	74 (23)	96 (27)	.240
Diabetes, *n* (%)	51 (8)	18 (6)	33 (9)	.087
Atrial fibrillation, *n* (%)	153 (22)	52 (16)	101 (28)	**<.001**
Prevalence of AMVP, *n* (%)	231 (34)	117 (36)	114 (32)	.33
VT at the ambulatory ECG monitor	203 (29)	105 (32)	98 (27)	.67
Frequent PVCs	38 (5)	19 (6)	19 (5)	1.00
Severe arrhythmic events	27 (4)	10 (3)	17 (5)	.20
Echocardiographic characteristics:				
MVP anatomy				.233
Flail, *n* (%)	43 (6)	13 (4)	30 (8)	
Bileaflet, *n* (%)	304 (45)	150 (46)	154 (43)	
Posterior, *n* (%)	241 (35)	115 (35)	126 (35)	
Anterior, *n* (%)	77 (11)	41 (13)	36 (10)	
MR				.002
None/trace or mild, *n* (%)	443 (66)	234 (73)	209 (60)	
Moderate, *n* (%)	166 (25)	61 (19)	105 (30)	
Severe, *n* (%)	61 (9)	27 (8)	34 (10)	
MAD presence, *n* (%)	282 (41)	153 (47)	129 (36)	**.006**
Absolute MAD length, mm	8.1 (3.0)	7.4 (2.6)	9.0 (3.2)	**<.001**
Indexed MAD				
Mean, mm	4.5 (1.6)	4.4 (1.5)	4.6 (1.7)	.33
Median, mm	4.3 (3.4–5.4)	4.2 (3.3–5.1)	4.4 (3.5–5.6)	.26
Indexed MAD > 4 mm, *n* (%)	127 (19)	64 (20)	63 (18)	.59
LV end-diastolic volume, mL/m2	61 (23)	54 (17)	67 (25)	**<.001**
LV end-systolic volume, mL/m2	24 (13)	20 (8)	27 (15)	**<.001**
LV mass, g/m2	86 (26)	77 (21)	94 (27)	**<.001**
LVEF, %	62 (8)	62 (8)	61 (8)	.05
LV GLS, %	−20 (4)	−20 (4)	−20 (4)	.192
MD, msec	60 (32)	60 (28)	60 (35)	.97
Right ventricular systolic function				.175
Normal	618 (93)	296 (94)	322 (92)	
Mildly reduced	36 (5)	12 (4)	24 (7)	
Moderately or more reduced	12 (2)	7 (2)	5 (1)	

Bold text indicates statistical significance (*P* < .05).

**Table 2:** Factors associated with MAD presence and length

	Presence of MAD				Length of MAD, mm			
	All patients (*N* = 682)				All patients with MAD (*N* = 282)			
	Univariable analysis		Multivariable analysis*		Univariable analysis		Multivariable analysis†	
	OR (95% CI)	*P* value	OR (95% CI)	*P* value	*β* ± SE	*P* value	*β* ± SE	*P* value

Age, per year increase*^†^	0.98 (0.97–0.99)	**<.001**	0.98 (0.97–0.99)	**<.001**	0.00 ± 0.01	.97	0.01 ± 0.01	.37
Sex, emale*^†^	1.56 (1.15–2.11)	**.005**	1.57 (1.13–2.19)	**.01**	−1.60 ± 0.36	**<.001**	− 0.81 ± 0.60	**.04**
Race, non-White*	0.63 (0.44–0.90)	**.01**	0.79 (0.53–1.18)	.25	0.25 ± 0.47	.59		
MR^†^								
No or mild	Reference				Reference			
Moderate	0.80 (0.56–1.16)	.24	1.12 (0.75–1.68)	.57	1.21 ± 0.43	.06		
Severe	0.44 (0.24–0.81)0.0080	**.01**	0.69 (0.35–1.28)0.2497	.25	1.65 ± 0.90	.07		
Bileaflet MVP*^†^	4.63 (3.34–6.42)	**<.001**	4.11 (2.93–5.79)	**<.001**	0.91 ± 0.40	**.02**	0.65 ± 0.41	.11
LVEDVi, per mL/m^2^ increase^†^	1.00 (1.00–1.01)	.27			0.03 ± 0.01	**.002**	0.01 ± 0.01	.28
LV mass, per g/m^2^ increase^†^	1.00 (0.99–1.00)	.40			0.04 ± 0.01	**.001**	0.01 ± 0.01	.77
LV GLS, per 1% decrease	1.04 (0.98–1.09)	.20			0.03 ± 0.07	.67		

*LVEDVi*, LV end-diastolic volume indexed.

Bold text indicates statistical significance.

* Multivariable model including age, sex, race, and bileaflet MVP.

† Multivariable model including age, sex, MR, bileaflet MVP, LVEDVi.

**Table 3:** Mitral morphofunctional predictors of AMVP

	Male patients				Female patients			
	Univariable analyses		Multivariable analyses		Univariable analyses		Multivariable analyses	
	OR (95% CI)	*P* value	OR (95% CI)	*P* value	OR (95% CI)	*P* value	OR (95% CI)	*P* value

Model 1: MAD presence								
Age, per year increase	1.00 (0.99–1.02)	.64	1.01 (1.00–1.03)	.18	0.99 (0.98–1.01)	.41	1.00 (0.98–1.01)	.56
Severe MR, yes or no	0.88 (0.40–1.91)	.7436	0.90 (0.39–1.94)	.79	1.76 (0.80–3.88)	.16	2.26 (0.98–5.18)	.06
Bileaflet MVP	1.15 (0.74–1.80)	.54	0.92 (0.55–1.54)	.76	1.82 (1.15–2.88)	**.01**	1.71 (1.05–2.82)	**.03**
MAD, yes or no	1.80 (1.14–2.84)	**.01**	2.12 (1.26–3.57)	**.01**	1.63 (1.03–2.57)	.04	1.47 (0.90–2.42)	.13
Model 2: MAD length indexed (categorical format)								
Age, per year	1.00 (0.99–1.02)	.64	1.01 (0.99–1.02)	.29	0.99 (0.98–1.01)	.41	0.99 (0.98–1.01)	.41
Severe MR, yes or no	0.88 (0.40–1.91)	.7436	0.97 (0.40–2.18)	.95	1.76 (0.80–3.88)	.16	2.87 (1.21–6.87)	**.02**
Bileaflet MVP	1.15 (0.74–1.80)	.54	1.01 (0.59–1.73)	.96	1.82 (1.15–2.88)	**.01**	1.67 (1.00–2.79)	**.05**
iMAD length								
Above median	2.00 (1.12–3.56)	**.02**	2.21 (1.15–4.25)	**.02**	1.45 (0.80–2.64)	.22	1.25 (0.65–2.38)	.50
Model 3: MAD l ength indexed (continuous format)								
Age, per year	1.00 (0.99–1.02)	.64	1.01 (0.99–1.02)	.29	0.99 (0.98–1.01)	.41	0.99 (0.98–1.01)	.36
Severe MR, yes or no	0.88 (0.40–1.91)	.7436	0.98 (0.41–2.19)	.96	1.76 (0.80–3.88)	.16	2.60 (1.11–6.19)	**.03**
Bileaflet MVP	1.15 (0.74–1.80)	.54	1.01 (0.59–1.72)	.96	1.82 (1.15–2.88)	**.01**	1.69 (1.02–2.83)	**.04**
iMAD, per mm (continuous)	1.13 (1.03–1.24)	**.01**	1.16 (1.04–1.29)	**.01**	1.10 (1.00–1.21)	.06	1.07 (0.96–1.18)	.21

Bold text indicates statistical significance (*P* < .05).

**Table 4:** Mediation analyses for the risk of AMVP

	Effect	95% CI	*P* value

Effect of bileaflet MVP on severe arrhythmia, MAD as a mediator in women			
ACME: indirect effect of MAD on arrhythmic MVP	0.004	−0.006 to 0.02	.39
ADE: direct effect of bileaflet MVP on arrhythmic MVP	0.04	0.006–0.08	**.04**
Proportion mediated	0.09	− 0.29 to 0.48	.38
Effect of bileaflet MVP on severe arrhythmia, MAD as a mediator in men			
ACME: indirect effect of MAD on arrhythmic MVP	0.02	0.01–0.05	**.002**
ADE: direct effect of bileaflet MVP on arrhythmic MVP		− 0.1 to 0.09	.16
Proportion mediated	0.03	0.11–1.51	**.03**

Bold text indicates statistical significance (*P* < .05).

*ACME*, Average causal mediation effect; *ADE*, average direct effect.

## HIGHLIGHTS

- MAD is more common in women but longer in men.
- Indexing MAD length to body size removes sex-based differences.
- Indexed MAD length predicts arrhythmic events in men but not in women.
- Bileaflet MVP increases arrhythmic risk in women independently of iMAD.
- Other factors may drive ventricular arrhythmias in women beyond MAD-related traction.

## Abbreviations

AMVP: Arrhythmic mitral valve prolapse
BSA: Body surface area
CMR: Cardiac magnetic resonance
ECG: Electrocardiogram
GLS: Global longitudinal strain
iMAD: Mitral annular disjunction indexed to body surface area
LA: Left atrial/atrium
LV: Left ventricular/ventricle
LVEF: Left ventricular ejection fraction
MAD: Mitral annular disjunction
MD: Mechanical dispersion
MR: Mitral regurgitation
MV: Mitral valve
MVP: Mitral valve prolapse
OR: Odds ratio
SCA: Sudden cardiac arrest
SCD: Sudden cardiac death
UCSF: University of California San Francisco
VT: Ventricular tachycardia
