# Supplementary material for: Sex-Based Differences in Mitral Annular Disjunction Severity and Arrhythmic Risk in Mitral Valve Prolapse
Source: J Am Soc Echocardiogr. Author manuscript; Available in PMC 2026 Jun 15. (PMC13267047; doi:10.1016/j.echo.2025.08.026)
Supplement: 1 [file NIHMS2182877-supplement-1.pdf]

Supplemental Table 1: Population characteristics stratified by arrhythmic MVP status

	**Non-Arrhythmic MVP (N=451, %)**	**Arrhythmic MVP (N=231, %)**	**p**
**Clinical Characteristics**			
Age, years	58 (17)	57.87 (16)	0.78
Sex, n (%)	242 (54)	114 (49)	0.33
Non-White race, n (%)	136 (30)	47 (20)	**0.01**
Body surface area, m^2	1.80 (0.3)	1.83 (0.3)	0.47
Hypertension, n (%)	190 (42)	66 (29)	**0.001**
Diabetes, n (%)	36 (8)	15 (7)	0.59
Atrial fibrillation, n (%)	87 (19)	66 (29)	**0.01**
Mitral regurgitation > moderate, n (%)	82 (19)	52 (23)	0.20
MAD, n (%)	166 (37)	116 (50)	**0.001**
Absolute MAD length, mm	8.0 (2.8)	8.3 (3.2)	0.41
Indexed MAD, mm	4.4 (1.5)	4.6 (1.7)	0.37
Indexed MAD > 4 mm, n (%)	75 (17)	52 (24)	0.06
LVEF, %	62 (8)	61 (8)	0.69
LVEDVi, ml/m^2	59 (23)	64 (22)	**0.01**
LVESVi, ml/m^2	23 (13)	25 (11)	0.13
LV-GLS, %	-20 (4)	-20 (4)	0.92
Mechanical Dispersion	59 (31)	65 (35)	0.13

Values are expressed as mean (SD) or absolute number (%). BSA = body surface area; LV = left ventricular; LVEDVi = left ventricular end-diastolic volume indexed; LVESVi = left ventricular end-systolic volume indexed; LVEF = left ventricular ejection fraction; LV-GLS = left ventricular global longitudinal strain; MAD = mitral annular disjunction; MADi = MAD indexed by BSA; MVP = mitral valve prolapse.

Supplemental Table 2: Morpho-functional predictors of arrhythmic mitral valve prolapse in the entire study population (not stratified by sex)

			**Model 1: MAD presence**		**Model 2: MAD length indexed (continuous format)**		**Model 2: MAD length indexed (continuous format)**	
	**Univariable analyses OR (95% CI)**	**p**	**Multivariable analyses #1 OR (95% CI)**	**p**	**Multivariable analyses #2 OR (95% CI)**	**p**	**Multivariable analyses #3 OR (95% CI)**	**p**
Age, per year increase	1.00 (0.99 – 1.01)	0.78	1.00 (0.99 – 1.01)	0.51	1.00 (0.99 – 1.01)	0.70	1.00 (0.99 – 1.01)	0.61
Sex	0.84 (0.61 – 1.16)	0.29	0.74 (0.52 – 1.06)	0.10	0.73 (0.51 – 1.04)	0.08	0.73 (0.51 – 1.05)	0.09
Severe MR	1.32 (0.89 – 1.95)	0.17						
Bileaflet MVP	1.45 (1.05 – 1.99)	**0.02**	1.14 (0.80 – 1.64)	0.47	1.23 (0.85 – 1.78)	0.27	1.22 (0.84 – 1.77)	0.29
MAD (yes or no)	1.73 (1.26 – 2.39)	**0.001**	1.64 (1.14 – 2.36)	**0.01**				
iMAD, per mm (cont.)	1.12 (1.05 – 1.20)	**0.001**			1.08 (1.00 – 1.17)	**0.04**		
iMAD length								
Below median	1.72 (1.13 – 2.60)	**0.01**					1.58 (0.99– 2.48)	0.05
Above median	1.72 (1.13 – 2.60)	**0.01**					1.45 (0.91 – 2.30)	0.12
Mechanical dispersion	1.01 (0.99 – 1.01)	0.13						
GLS	1.00 (0.94 – 1.07)	0.92						
LVEDVi	1.01 (1.00 – 1.02)	**0.01**	1.01 (1.00 – 1.02)	**0.01**	1.01 (1.00 – 1.02)	**0.01**	1.01 (1.00 – 1.02)	**0.01**
LVEF	1.00 (0.98 – 1.02)	0.69						
LAESVi	1.00 (1.00 – 1.01)	0.29						
TWI	1.10 (0.77 – 1.56)	0.60						

CI = confidence interval; EF = ejection fraction; GLS = global longitudinal strain; iMAD = mitral annular disjunction indexed to body surface area; LAESVi = left atrium end systolic volume indexed: LVEDVi = left ventricle end diastolic volume indexed; MR = mitral regurgitation; MVP = mitral valve prolapse; OR = odds ratio; TWI = T wave inversion

Supplemental Table 3. Covariate balance between male and female patients after propensity score matching on hypertension, atrial fibrillation, indexed LV end-diastolic volume, indexed LV end-systolic volume, and LV mass index.

Variable	Treated Mean	Control Mean	Std. Mean Diff	Variance Ratio	eCDF Mean	eCDF Max	Std. Pair Distance
Distance	0.4977	0.4933	0.0247	1.0339	0.0075	0.0341	0.0270
Hypertension (No)	0.6364	0.6420	-0.0125	—	0.0057	0.0057	0.8130
Hypertension (Yes)	0.3636	0.3580	0.0125	—	0.0057	0.0057	0.8130
AF (No)	0.8011	0.8182	-0.0469	—	0.0170	0.0170	0.8284
AF (Yes)	0.1989	0.1818	0.0469	—	0.0170	0.0170	0.8284
LVEDVI	56.76	57.20	-0.0286	1.0081	0.0104	0.0341	0.7515
LVESVI	21.50	21.80	-0.0395	1.2190	0.0111	0.0341	0.8174
LV Mass Index	82.69	83.56	-0.0417	0.8815	0.0169	0.0625	0.7940

Standardized mean differences (SMD) were all well below 0.1, indicating excellent post-matching balance. Standardized pairwise distances (Std. Pair Dist.) further support effective matching.

AF = atrial fibrillation; LV = left ventricle; LVEDVI = left ventricular end-diastolic volume indexed; LVESVI = left ventricular end-systolic volume indexed

Supplemental Table 4: Morpho-functional predictors of arrhythmic mitral valve prolapse

	Male Patients				Female Patients					
	**Univariable analyses OR (95% CI)**	**p**	**Multivariable analyses OR (95% CI)**	**p**	**Univariable analyses OR (95% CI)**	**p**	**Multivariable analyses #1 OR (95% CI)**	**p**	**Multivariable analyses #2 OR (95% CI)**	**p**
**Model 1: MAD presence**										
Age, per year increase	1.00 (0.99 – 1.02)	0.64	1.01 (1.00 – 1.03)	0.14	0.99 (0.98 – 1.01)	0.41	0.99 (0.98 – 1.01)	0.37	1.00 (0.97 – 1.02)	0.82
Severe MR	1.39 (0.82 – 2.34)	0.22	1.25 (0.69 – 2.23)	0.46	1.29 (0.71 – 2.35)	0.40	1.63 (0.85 – 3.09)	0.14	1.52 (0.56 – 3.88)	0.39
Bileaflet MVP	1.15 (0.74 – 1.80)	0.54	1.00 (0.58 – 1.71)	0.99	1.82 (1.15 – 2.88)	**0.01**	1.69 (1.02 – 2.82)	**0.04**	0.92 (0.41 – 2.05)	0.84
MAD (yes or no)	1.80 (1.14 – 2.84)	**0.01**	2.08 (1.22 – 3.56)	**0.01**	1.63 (1.03 – 2.57)	**0.04**	1.06 (0.96 – 1.17)	0.27	0.95 (0.42 – 2.11)	0.90
Mechanical dispersion	1.00 (0.99 – 1.01)	0.76			1.02 (1.00 – 1.03)	**0.01**			1.02 (1.00 – 1.03)	**0.03**
GLS	0.99 (0.90 – 1.08)	0.77			1.02 (0.93 – 1.12)	0.64				
LVEDVi	1.01 (1.00 – 1.02)	**0.04**	1.01 (0.99 – 1.02)	0.06	1.02 (1.00 – 1.03)	**0.02**			1.01 (0.98 – 1.04)	0.67
LVEF	1.00 (0.97 – 1.02)	0.74			0.99 (0.97 – 1.02)	0.72				
LAESVi	1.01 (0.99 – 1.02)	0.12			1.00 (0.98 – 1.01)	0.85				
TWI	0.92 (0.56 – 1.50)	0.74			1.35 (0.81 – 2.24)	0.25				
**Model 2: MAD length indexed (categorical format)**										
Age, per year	1.00 (0.99 – 1.02)	0.64	1.01 (1.00 – 1.03)	0.17	0.99 (0.98 – 1.01)	0.41	0.99 (0.98 – 1.01)	0.40	1.00 (0.97 – 1.02)	0.80
Severe MR	1.39 (0.82 – 2.34)	0.22	1.18 (0.64 – 2.15)	0.59	1.29 (0.71 – 2.35)	0.40	1.80 (0.93 – 3.47)	0.08	1.72 (0.61 – 4.57)	0.29
Bileaflet MVP	1.15 (0.74 – 1.80)	0.54	1.09 (0.62 – 1.90)	0.77	1.82 (1.15 – 2.88)	**0.01**	1.68 (1.01 – 2.81)	**0.04**	0.89 (0.39 – 1.99)	0.78
iMAD length										
Below median	1.45 (0.76 – 2.76)	0.26	1.63 (0.80 – 3.25)	0.17	1.83 (1.05 – 3.20)	0.03	1.71 (0.94 – 3.11)	0.08	1.33 (0.48 – 3.52)	0.57

Above median	2.00 (1.12 – 3.56)	**0.02**	2.06 (1.05 – 4.02)	**0.03**	1.45 (0.80 – 2.64)	0.22	1.17 (0.61 – 2.22)	0.63	0.77 (0.26 – 2.09)	0.63
Mechanical dispersion	1.00 (0.99 – 1.01)	0.76			1.02 (1.00 – 1.03)	**0.01**			1.02 (1.00 – 1.03)	**0.02**
GLS	0.99 (0.90 – 1.08)	0.77			1.02 (0.93 – 1.12)	0.64				
LVEDVi	1.01 (1.00 – 1.02)	**0.04**	1.01 (1.00 – 1.02)	0.11	1.02 (1.00 – 1.03)	**0.02**			1.01 (0.98 – 1.04)	0.58
LVEF	1.00 (0.97 – 1.02)	0.74			0.99 (0.97 – 1.02)	0.72				
LAESVi	1.01 (0.99 – 1.02)	0.12			1.00 (0.98 – 1.01)	0.85				
TWI	0.92 (0.56 – 1.50)	0.74			1.35 (0.81 – 2.24)	0.25				
**Model 3: MAD length indexed (continuous format)**										
Age, per year	1.00 (0.99 – 1.02)	0.64	1.01 (1.00 – 1.03)	0.18	0.99 (0.98 – 1.01)	0.41	0.99 (0.98 – 1.01)	0.370	1.00 (0.97 – 1.02)	0.81
Severe MR	1.39 (0.82 – 2.34)	0.22	1.18 (0.64 – 2.15)	0.59	1.29 (0.71 – 2.35)	0.40	1.63 (0.85 – 3.09)	0.14	1.54 (0.57 – 3.93)	0.37
Bileaflet MVP	1.15 (0.74 – 1.80)	0.54	1.11 (0.64 – 1.93)	0.70	1.82 (1.15 – 2.88)	**0.01**	1.69 (1.02 – 2.82)	**0.04**	0.90 (0.39 – 2.00)	0.79
iMAD, per mm (cont.)	1.13 (1.03 – 1.24)	**0.01**	1.13 (1.01 – 1.27)	**0.03**	1.10 (1.00 – 1.21)	0.06	1.06 (0.96 – 1.17)	0.27	0.98 (0.83 – 1.15)	0.85
Mechanical dispersion	1.00 (0.99 – 1.01)	0.76			1.02 (1.00 – 1.03)	**0.01**			1.02 (1.00 – 1.03)	**0.03**
GLS	0.99 (0.90 – 1.08)	0.77			1.02 (0.93 – 1.12)	0.64				
LVEDVi	1.01 (1.00 – 1.02)	**0.04**	1.01 (1.00 – 1.02)	0.12	1.02 (1.00 – 1.03)	**0.02**			1.01 (0.98 – 1.04)	0.60
LVEF	1.00 (0.97 – 1.02)	0.74			0.99 (0.97 – 1.02)	0.72				
LAESVi	1.01 (0.99 – 1.02)	0.12			1.00 (0.98 – 1.01)	0.85				
TWI	0.92 (0.56 – 1.50)	0.74			1.35 (0.81 – 2.24)	0.25				

CI = confidence interval; EF = ejection fraction; GLS = global longitudinal strain; iMAD = mitral annular disjunction indexed to body surface area; LAESVi = left atrium end systolic volume indexed: LVEDVi = left ventricle end diastolic volume indexed; MR = mitral regurgitation; MVP = mitral valve prolapse; OR = odds ratio; TWI = T wave inversion
